# Overexpression of a WRKY transcription factor McWRKY57-like from *Mentha canadensis* L. enhances drought tolerance in transgenic Arabidopsis

**DOI:** 10.1186/s12870-023-04213-y

**Published:** 2023-04-25

**Authors:** Yang Bai, Ting Zhang, Xiaowei Zheng, Bingxuan Li, Xiwu Qi, Yu Xu, Li Li, Chengyuan Liang

**Affiliations:** 1 Jiangsu Key Laboratory for the Research and Utilization of Plant Resources, Institute of Botany, Jiangsu Province and Chinese Academy of Sciences (Nanjing Botanical Garden Mem. Sun Yat-Sen), Nanjing, 210014 China; 2 The key laboratory of quality improvement of agriculture products of Zhejiang province, college of advanced agriculture sciences, Zhejiang A&F University, Hangzhou, 311300 China

**Keywords:** Transcription factor, McWRKY57-like, *Mentha canadensis*, Drought tolerance

## Abstract

**Background:**

Drought has become a major environmental problem affecting crop production. Members of the WRKY family play important roles in plant development and stress responses. However, their roles in mint have been barely explored.

**Results:**

In this study, we isolated a drought-inducible gene *McWRKY57-like* from mint and investigated its function. The gene encodes a group IIc WRKY transcription factor, McWRKY57-like, which is a nuclear protein with a highly conserved WRKY domain and a C2H2 zinc-finger structure, and has transcription factor activity. Its expression levels were examined in different tissues of mint and under the treatment of mannitol, NaCl, abscisic acid, and methyl jasmonate. We found that *McWRKY57-like* overexpression in Arabidopsis significantly increased drought tolerance. Further studies showed that under drought stress, *McWRKY57-like*-overexpressing plants had higher chlorophyll, soluble sugar, soluble protein, and proline contents but lower water loss rate and malondialdehyde content than wild-type plants. Moreover, the activities of antioxidant enzymes catalase, superoxide dismutase, and peroxidase were enhanced in *McWRKY57-like* transgenic plants. Furthermore, qRT-PCR analysis revealed that the drought-related genes *AtRD29A*, *AtRD29B*, *AtRD20*, *AtRAB18*, *AtCOR15A*, *AtCOR15B*, *AtKIN2*, and *AtDREB1A* were upregulated in *McWRKY57-like* transgenic plants than in wild-type Arabidopsis under simulated drought conditions.

**Conclusion:**

These data demonstrated that *McWRKY57-like* conferred drought tolerance in transgenic Arabidopsis by regulating plant growth, osmolyte accumulation and antioxidant enzyme activities, and the expression of stress-related genes. The study indicates that *McWRKY57-like* plays a positive role in drought response in plants.

**Supplementary Information:**

The online version contains supplementary material available at 10.1186/s12870-023-04213-y.

## Background

Drought is a detrimental event caused by chronic water shortage. It restricts plant distribution, growth, and survival, thereby adversely affecting agricultural production [1]. To adapt to drought stress, plants have evolved many mechanisms to alter and regulate various physiological and biochemical processes at the molecular, cellular, biochemical, and physiological levels [2, 3]. At the molecular level, drought tolerance involves multiple genes associated with cellular signaling pathways. Transcriptional regulation of genes plays a crucial role in plant responses to drought stress. Transcription factors (TFs), such as bHLH (basic helix-loop-helix protein), MYB (Myeloblastosis), NAC (NAM, ATAF1/2, and CUC2), DREB (Dehydration Responsive Element-Binding Protein), bZIP (basic region/leucine Zipper), and WRKY, regulate target gene expression during plant drought stress response by binding to DNA binding domains on the promoters of the target genes [4–9].

WRKY is one of the largest TF families in plants. It is defined by a highly conserved WRKYGQK motif at the N-terminus and a zinc-finger-like motif at the C-terminus and generally binds to the specific DNA motif W-box [TGACC(A/T)]. Based on the number of DNA binding domains and the features of the zinc-finger-like motif, WRKY TFs are classified into three groups: Group I, Group II, and Group III, wherein Group II is further divided into five subgroups (IIa, IIb, IIc, IId, and IIe) [10, 11]. Since the first identification of the WRKY gene *SPF1* from sweet potato [12], WRKY genes have been cloned from other plant species, including Arabidopsis [13], rice [14], wheat [15], *Artemisia annua* [16], *Catharanthus roseus* [17], *Taxus chinensis* [18], and *Salvia miltiorrhiza* [19], and have attracted more and more attention. WRKY genes have been shown to play pivotal roles in plant development and response to various stresses, such as cold, drought, salt, and pathogens, by activating or preventing the expression of their target genes [20, 21]. Recent studies have further confirmed the key roles of WRKYs in the stress responses of various plant species. Overexpression of *SlWRKY8* in tomato increases its resistance to pathogen *Pseudomonas syringae* pv. tomato DC3000 (Pst. DC3000) [22]. Grape VvWRKY30 plays a positive role under salinity stress [23]. *ZmWRKY79* gene positively regulates the drought tolerance of maize [24]. Overexpression of *ZmWRKY106* improves the tolerance of transgenic Arabidopsis to high temperatures [25]. Therefore, WRKYs are considered important regulators of the stress responses and require further investigation.

Mint (*Mentha canadensis* L.), a widely distributed perennial herb in the family Lamiaceae in China, has been long regarded as a traditional Chinese medicinal material for its rich essential oil components. It is cultivated for its medicinal and aroma properties, and is widely used in food, cosmeceutical, personal hygiene, and pharmaceutical industries, as well as in clinics for the treatment of neurological, respiratory, reproductive, and digestive disorders [26]. Drought stress significantly reduces mint growth and essential oil yield and affects the abundance of essential oil components (menthol, menthofuran, plugene, etc.) [27]. However, the mechanisms underlying mint’s response to drought stress have been barely explored.

The WRKY TF WRKY57 has been well studied in Arabidopsis. It plays a critical role in regulating the response to drought [28, 29], leaf senescence [30], and pathogenic resistance [31]. Recent studies have shown that WRKY family members MsGSW2 and MhGSW2 from mint cultivars *M. spicata* and *M. haplocalyx* regulate glandular trichome development in transgenic *A. annua* [32]. However, very few WRKY TFs associated with drought tolerance in mint have been characterized, and the underlying molecular mechanisms are largely unknown. In this study, we cloned a *WRKY57* orthologous gene named *McWRKY57-like* and analyzed its transcriptional patterns and subcellular distribution. Moreover, we conducted phenotypic and physiological index analyses and found that overexpression of the *McWRKY57-like* gene in Arabidopsis markedly enhanced drought tolerance. To further elucidate the molecular mechanisms by which the *McWRKY57-like* gene enhances stress tolerance, we examined the expression levels of multiple relevant factors in wild-type and *McWRKY57-like* overexpression plants under normal and simulated drought conditions using qRT-PCR analyses. Our results indicate that McWRKY57-like plays a positive role in regulating drought tolerance in plants and can be an important candidate gene in breeding new cultivars with excellent drought tolerance.

## Results

### ***McWRKY57-like*** isolation and sequence analysis

**Fig. 1:**
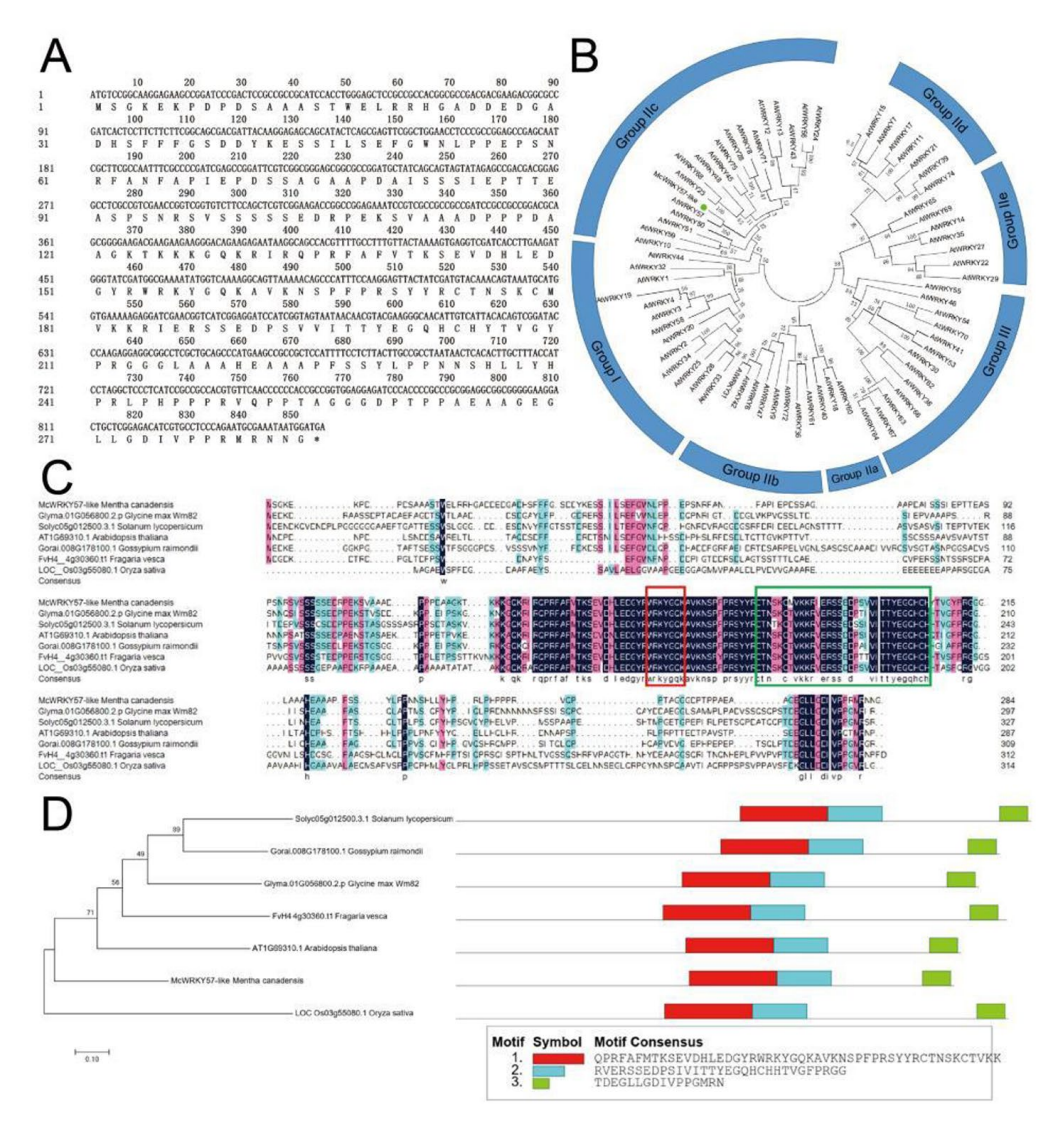
Bioinformatics analysis of *McWRKY57-like*. **A** Nucleotide sequence and amino acid sequence of *McWRKY57-like*. **B** Phylogenetic analysis of McWRKY57-like and Arabidopsis WRKYs. McWRKY57-like is marked with a green solid circle. **C** Sequence alignment of McWRKY57-like to other plant WRKY57 homologous proteins. The WRKYGQK conservative sequence was outlined by a red box and the C2H2-type zinc finger motif was outlined by a green box. **D** Phylogenetic relationship and motif patterns of McWRKY57-like and other plant WRKY57 homologous proteins

Previous studies have confirmed that WRKY57 positively regulates drought tolerance in Arabidopsis [28, 29]. To identify putative WRKY genes related to drought tolerance in mint, we performed a BLASTP search (https://blast.ncbi.nlm.nih.gov/Blast.cgi) on the reported transcriptome data (SRP132644) at the National Center for Biotechnology Information (NCBI) [33] using the AtWRKY57 amino acid sequence as a query. A *WRKY57* homologous gene with an open reading frame of 855 codons and encoding 284 amino acid residues was screened out from *M. canadensis* L. and named *McWRKY57-like* (Fig. 1A). Phylogenetic tree analysis of Arabidopsis WRKY family proteins showed that McWRKY57-like clustered with AtWRKY57, which belongs to the IIc subgroup of the WRKY family (Fig. 1B). Sequence alignment of McWRKY57-like with other WRKY57 homologous proteins from Arabidopsis, *Glycine max*, *Solanum lycopersicum*, *Gossypium raimondii*, *Fragaria vesca*, and *Oryza sativa* showed that McWRKY57-like contains a highly conserved WRKYGQK domain and a C2H2-type (C-X_4–5_-C-X_22–23_-H-X_1_-H) zinc finger motif (Fig. 1 C and D), indicating that McWRKY57-like has a close relationship with the WRKY57 ortholog of *Arabidopsis thaliana* (Fig. 1D).

### Subcellular localization and transcriptional activity of McWRKY57-like

**Fig. 2:**
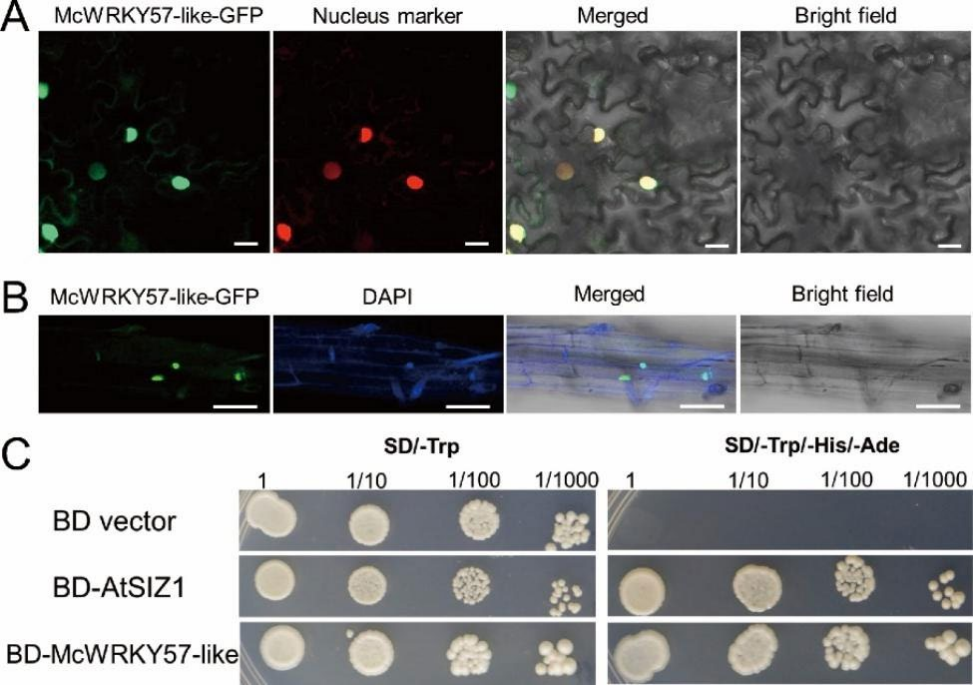
Subcellular localization of McWRKY57-like. **A** McWRKY57-like-GFP fusion protein was transiently co-expressed with nucleus marker in tobacco leave epidermal cells. Bar = 20 μm. **B** McWRKY57-like-GFP fusion protein was expressed in transgenic Arabidopsis roots and stained with DAPI. Bar = 50 μm. **C** Transcriptional activation verification of McWRKY57-like. BD-McWRKY57-like, the experimental group; BD-AtSIZ1, positive control; BD vector, the empty control

To determine the subcellular localization of McWRKY57-like, we constructed the *35 S::McWRKY57-like-GFP* plasmid, in which *McWRKY57-like* was fused to the N-terminus of the green fluorescent protein (GFP) gene under the control of the 35 S promoter. Then, the recombinant vector was transiently co-expressed in *Nicotiana benthamiana* leaves with a nuclear marker *35 S::D53-RFP* [34]. The *35 S::McWRKY57-like-GFP* construct was further transformed into Arabidopsis wild-type (WT) plants, and the subcellular location of McWRKY57-like in the transgenic seedlings was observed under a confocal microscope after DAPI staining. The observations reveal that McWRKY57-like is expressed in the nucleus (Fig. 2A and B).

We further investigated the transcriptional activation activity of McWRKY57-like using a yeast assay system. The full-length coding sequence of *McWRKY57-like* was introduced into the *pGBKT7* vector to generate the *BD-McWRKY57-like*, which was then transformed into yeast strain Y2H. The yeast strains containing *pGBKT7* (BD vector) and *pGBKT7-AtSIZ1* (BD-AtSIZ1) were used as the empty and positive controls, respectively. As shown in Fig. 2C, all yeasts in our experiment grew normally on SD/-Trp medium, but only yeast transformed with the BD-McWRKY57-like or BD-AtSIZ1 could grow normally on SD/-Trp/-His/-Ade medium. These results confirm that McWRKY57-like has transcription factor activity.

### Analysis of ***McWRKY57-like*** gene expression in different tissues

**Fig. 3:**
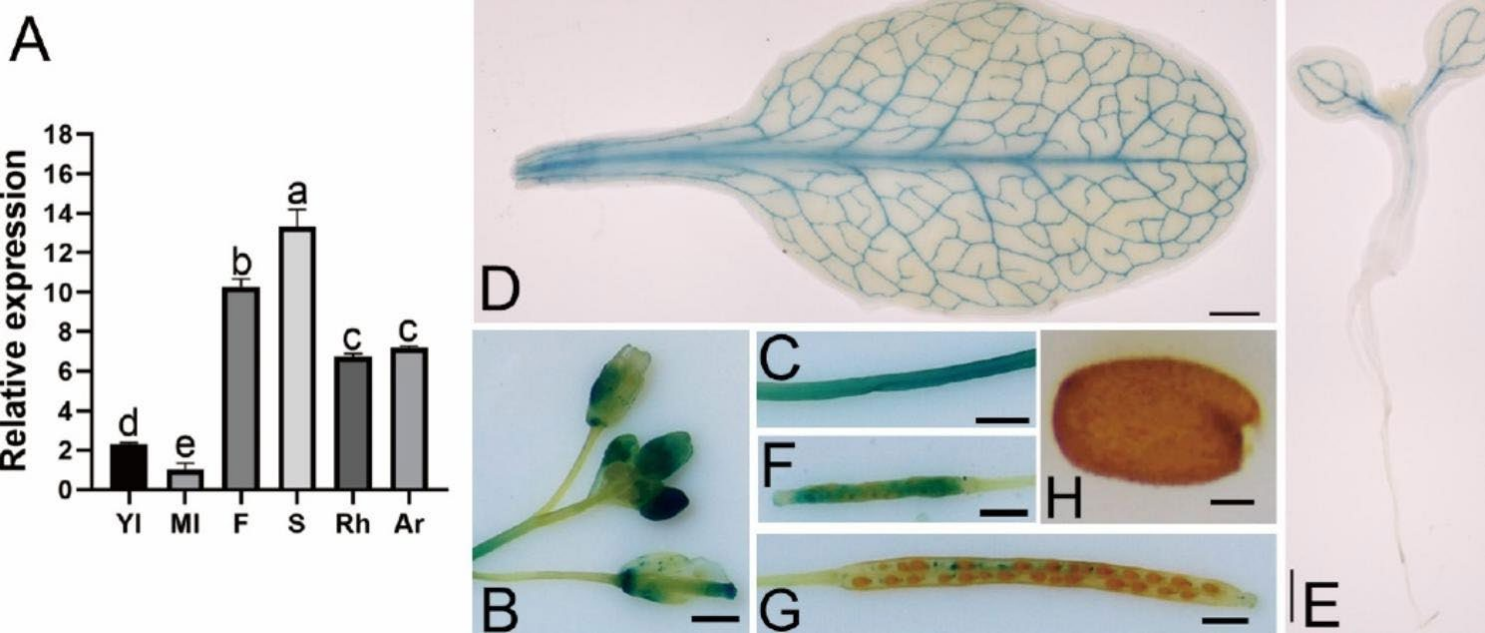
Tissue expression of *McWRKY57-like* in *M. canadensis* and Arabidopsis thaliana. **A** Expression profiles of the McWRKY57-like gene in *M. canadensis*. Yl, young leaves; Ml, mature leaves; F, flowers; S, stems; Rh, rhizomes, Ar, adventitious roots. Total RNA was extracted from different tissues of *M. canadensis* plants for qRT-PCR. Values were standardized using the *M. canadensis McACT* gene. Values are the means of three independent experiments ± standard error (SE) (n = 3). **B-H** Analysis of *McWRKY57-like* expression in transgenic Arabidopsis plants by GUS staining. **B** GUS staining in flowers. Bar = 1 mm. **C** GUS staining in stems. Bar = 1 mm. **D** GUS staining in rosette leaves. Bar = 1 mm. **E** GUS staining in a 7-day-old seedling grown on agar. Bar = 1 mm. **F** GUS staining in 3-DAP siliques. DAP, day after pollination. Bar = 1 mm. **G** GUS staining in 10-DAP siliques. Bar = 1 mm. **H**. GUS staining in mature seeds. Bar = 100 μm

To investigate the potential working locations of the *McWRKY57-like* gene in *M. canadensis*, we detected *McWRKY57-like* transcript levels in different tissues, including young leaves, mature leaves, flowers, stems, rhizomes and adventitious roots using quantitative reverse transcription polymerase chain reaction (qRT-PCR). The results showed that the *McWRKY57-like* gene was expressed the most in stems, followed by flowers, adventitious roots, rhizomes, young leaves, and mature leaves in descending order (Fig. 3A). To further determine the *McWRKY57-like* expression pattern, we constructed a GUS vector driven by the *McWRKY57-like* promoter, which was obtained using Genome Walking Kit (Figure S1), and introduced it into WT Arabidopsis plants. One transgenic *Pro*_*McWRKY57−like*_-*GUS* line was selected to analyze the expression of the *McWRKY57-like* gene. GUS expression was detectable by histochemical staining in young seedlings, rosette leaves, stems, flowers, young siliques, and mature siliques, but not in mature seeds of the transgenic Arabidopsis (Fig. 3B–H), showing the strongest GUS staining in flowers and stems (Fig. 3B and C). GUS expression was also detectable in rosette leaves and cotyledons of young seedlings, where only veins exhibited GUS signal (Fig. 3D and E). Unexpectedly, GUS staining was undetectable in the roots of transgenic Arabidopsis seedlings. Interestingly, GUS expression was higher in young siliques (about 3 days after pollination) than in mature siliques (about 10 days after pollination) and was not detectable in mature seeds (Fig. 3F–H). These results suggest that *McWRKY57-like* is significantly expressed in flowers, stems, adventitious roots, and rhizomes of mint, showing a different pattern from transgenic Arabidopsis plants.

### ***McWRKY57-like*** expression under different treatments

**Fig. 4:**
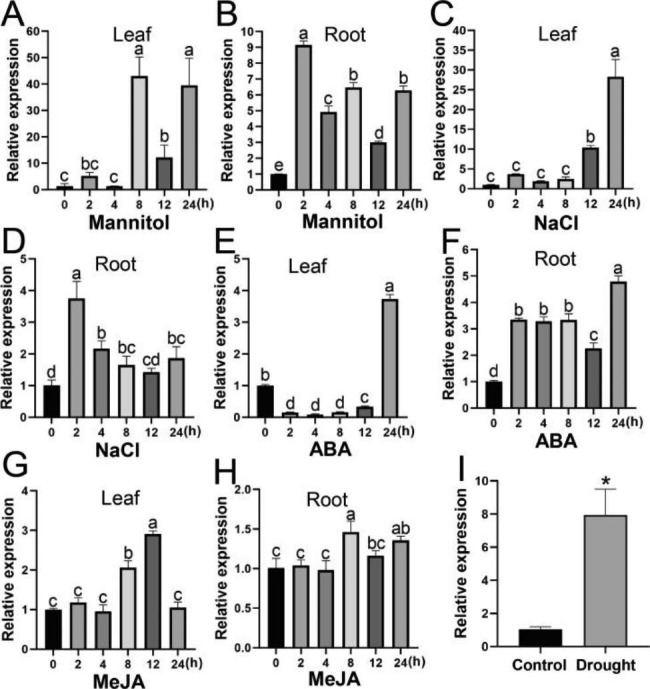
Expression Analysis of the *McWRKY57-like* gene under different treatments. **A** and **B** Time-course expression levels of *McWRKY57-like* in leaves and adventitious roots under treatment with 300 mM mannitol. **C** and **D** Time-course expression levels of *McWRKY57-like* in leaves and adventitious roots under treatment with 150 mM NaCl. **E** and **F** Time-course expression levels of *McWRKY57-like* in leaves and adventitious roots under treatment with 100 µM ABA. **G** and **H** Time-course expression levels of *McWRKY57-like* in leaves and adventitious roots under treatment with 200 µM MeJA. For each treatment, *McACT* was used as the reference gene, and expression values are relative to that of 0 h, and data represented means ± SE of three replicates. Different letters indicate significant differences at *P* < 0.05, as determined by one-way analysis of variance (ANOVA) with Tukey’s post-test. **I** Expression profiles of the *McWRKY57-like* gene in leaves of *M. canadensis* under natural drought stress. *McACT* was used as the reference gene, and the expression level was normalized to the untreated control. The asterisk (*) indicated significant differences at *P* < 0.05, as determined by one-way analysis of variance (ANOVA) with Tukey’s post-test

Genes in the WRKY family play pivotal roles in plant response to abiotic stresses, and their expression levels are influenced by environmental factors and hormones [20]. To further analyze the potential functions of the *McWRKY57-like* gene, we performed qRT-PCR to examine its expression level in leaves and adventitious roots after mannitol, NaCl, abscisic acid (ABA), and methyl jasmonate (MeJA) treatments. The results showed that (1) when treated with 300 mM mannitol, *McWRKY57-like* expression was markedly induced in leaves at 8, 12, and 24 h after treatment (h.a.t), reaching a peak at 8 h.a.t at about a 33-fold increase, and it was increased in adventitious roots by about 2–8-fold within 2–24 h, reaching a peak at 2 h.a.t (Fig. 4A and B); (2) under treatment with 150 mM NaCl, *McWRKY57-like* expression was increased in leaves within 2–24 h.a.t, reaching a peak at 24 h.a.t at about a 27-fold increase, and increased in adventitious roots by about 1–2-fold at 2, 4, 8, and 24 h.a.t while did not change too much in 12 h.a.t (Fig. 4C and D); (3) when treated with ABA, *McWRKY57-like* expression decreased in leaves at 2–12 h.a.t. while increased observably by almost 3 fold at 24 h.a.t., and enhanced remarkably in adventitious roots at each time point, reaching a peak by about 4 fold at 24 h.a.t. (Fig. 4E and F); and (4) Under the MeJA treatment, *McWRKY57-like* expression increased significantly in leaves at 8 and 12 h.a.t. and enhanced in adventitious roots at 8 and 24 h.a.t. (Figs. 4G and H). Among these four treatments, mannitol treatment induced *McWRKY57-like* expression to the highest level in both leaves and adventitious roots. We also examined *McWRKY57-like* expression in leaves under natural drought stress and found that its mRNA level was increased by nearly 7-fold compared with the control (Fig. 4I). These results suggest that the *McWRKY57-like* gene responds most intensely to drought stress and may play a role in regulating drought stress in plants.

### Overexpression of ***McWRKY57-like*** in Arabidopsis increases drought tolerance

**Fig. 5:**
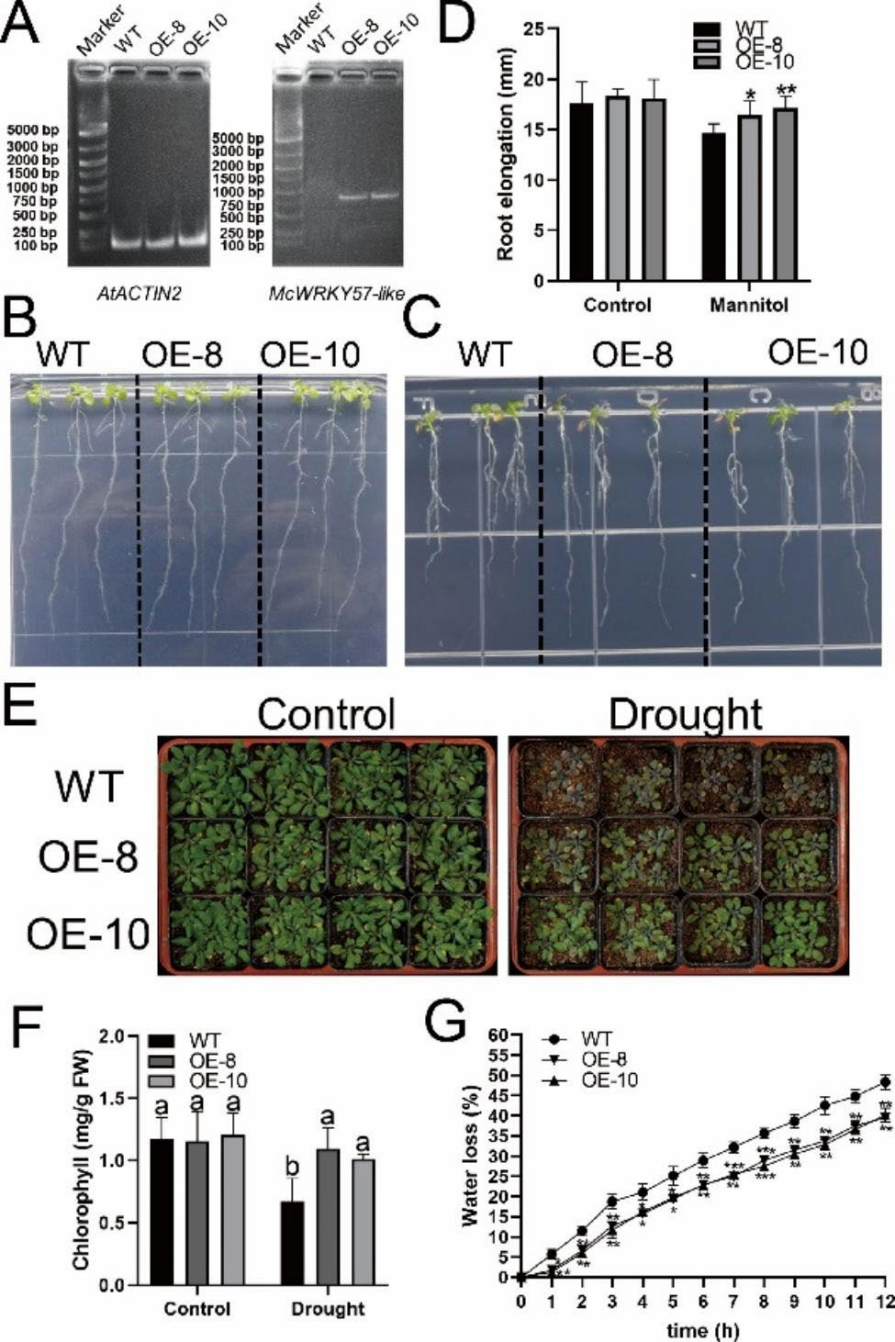
Overexpression of the *McWRKY57-like* gene improved drought tolerance in transgenic Arabidopsis plants. **A** Level of *McWRKY57-like* transcript in 7-day-old seedlings of WT, and *McWRKY57-like* overexpression (OE) lines (OE-8 and OE-10). **B** and **C** Phenotypes of 11-day-old seedlings of WT and transgenic lines OE-8 and OE-10 under control conditions and treatment with 300 mM mannitol, respectively. **D** Comparison of primary root elongation between seedlings of WT and transgenic lines OE-8 and OE-10 under control conditions and treatment with 300 mM mannitol. Data are means of the 20 replicates with SE. Student’s *t* tests were performed to show significant differences in primary root elongation between OE and WT lines. *P < 0.05, **P < 0.01. **E** The growth status of transgenic and WT plants under control and drought conditions. **F** Measurement of chlorophyll content of leaves from transgenic and WT plants under control and natural drought conditions. Data represented means ± SE of three replicates. Different letters indicate significant differences at *P* < 0.05, as determined by one-way analysis of variance (ANOVA) with Tukey’s post-test. **G** Water loss rate of detached leaves from transgenic and WT plants under given conditions. Data are means of the 3 replicates with SE. Student’s *t* tests were performed to show significant differences in water loss rate between OE and WT lines. *P < 0.05, **P < 0.01, ***P < 0.001

To further investigate whether *McWRKY57-like* contributes to drought resistance, the *35 S::McWRKY57-like-GFP* construct was transformed into Arabidopsis WT plants. Two homozygous *McWRKY57-like* overexpression (OE) lines, *McWRKY57-like* OE-8 and OE-10, were obtained, and *McWRKY57-like* expression was assessed in transgenic seedlings using RT-PCR (Fig. 5A). We next performed drought treatments for plants at seedling stage. We observed no significant difference between WT and *McWRKY57-like OE* lines grown on 1/2 MS without 300 mM mannitol treatment (Fig. 5B and D). In contrast, we observed longer roots in *McWRKY57-like OE* lines than in WT plants grown on 1/2 MS with 300 mM mannitol (Fig. 5C and D).

To further investigate the function of *McWRKY57-like* in plant vegetative growth, WT and transgenic plants grown in soils for 3 weeks were subjected to natural drought stress. After 10 days of natural drought treatment, most leaves maintained green in OE-8 and OE-10 transgenic lines but withered and chlorotic in WT plants (Fig. 5E). We further measured leaf chlorophyll content in WT and *McWRKY57-like OE* lines. We found no significant difference in leaf chlorophyll content between WT and *McWRKY57-like OE* lines under control conditions. However, the leaf chlorophyll content was significantly higher in *McWRKY57-like OE* plants than in WT plants after natural drought treatment (Fig. 5F). Reducing water loss is advantageous in enhancing drought tolerance. Therefore, we analyzed leaf water loss rates in WT and *McWRKY57-like OE* plants. The results showed that both *McWRKY57-like OE* lines (OE-8 and OE-10) exhibited evidently lower water loss rates than WT under given conditions (Fig. 5G). These results suggest that *McWRKY57-like* overexpression enhances drought tolerance of transgenic Arabidopsis.

### ***McWRKY57-like*** overexpression in Arabidopsis improves osmolyte accumulation and antioxidant enzyme activities

**Fig. 6:**
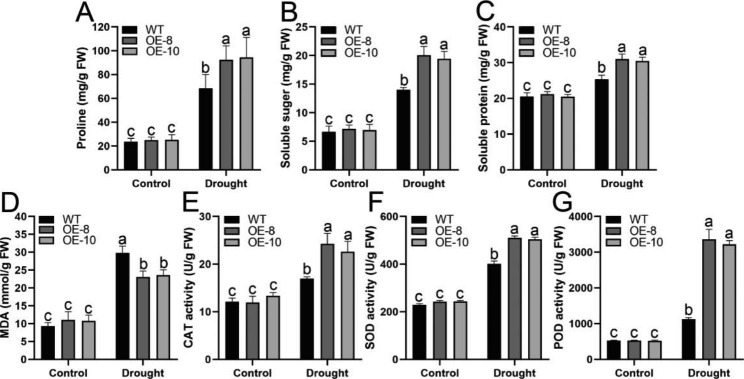
Overexpression of the *McWRKY57-like* gene improved osmolyte accumulation and antioxidant enzyme activities in transgenic Arabidopsis plants. **A-C** Proline, soluble sugar and soluble protein contents of WT and *McWRKY57-like* OE lines (OE-8 and OE-10) under normal and natural drought conditions. **D** MDA content of WT and *McWRKY57-like* OE lines (OE-8 and OE-10) under normal and natural drought conditions. **E-G** CAT, SOD and POD activities in leaves of WT and *McWRKY57-like* OE lines (OE-8 and OE-10) under normal and natural drought conditions. Data are means of the 3 replicates with SE. Different letters indicate significant differences at *P* < 0.05, as determined by one-way analysis of variance (ANOVA) with Tukey’s post-test

Drought stress can cause osmotic stress in plants. Under osmotic stress, plants can synthesize some osmotic-regulating substances. To further elucidate the mechanism by which *McWRKY57-like* enhances drought tolerance, the contents of proline, soluble sugar, and soluble protein were detected in WT and *McWRKY57-like OE* plants under normal and drought conditions. We found that the contents of proline, soluble sugar, and soluble protein were not evidently different between WT and *McWRKY57-like OE* plants under control conditions but were significantly increased in both WT and *McWRKY57-like OE* plants under drought stress. Furthermore, these increases were more pronounced in *McWRKY57-like OE* lines (Fig. 6A–C). These results indicate that *McWRKY57-like* overexpression enhances the osmoregulation capacity of transgenic plants under drought stress.

Drought stress can also induce the accumulation of reactive oxygen species (ROS), such as superoxide anion radicals (O_2_^−^) and hydrogen peroxide (H_2_O_2_), leading to oxidative damage to plant cells [35]. Malondialdehyde (MDA) is a product of ROS-stimulated lipid peroxidation, and MDA content is commonly used to assess the extent of ROS-mediated damage to plants [36]. We examined MDA content in plants under control and drought conditions. The results showed that MDA content increased in WT and *McWRKY57-like OE* plants after drought treatment, and the increase was remarkably higher in WT than in *McWRKY57-like OE* lines (Fig. 6D). We also tested the enzymic activities of catalase (CAT), superoxide dismutase (SOD), and peroxidase (POD), the key enzymes for scavenging ROS. The results showed that their activities were increased more evidently under drought stress in *McWRKY57-like OE* plants than in WT plants (Fig. 6E–G). These results suggest that *McWRKY57-like* overexpression enhances the activities of antioxidant enzymes in transgenic plants under drought stress.

### ***McWRKY57-like*** overexpression upregulates the expression of the stress-responsive genes

**Fig. 7:**
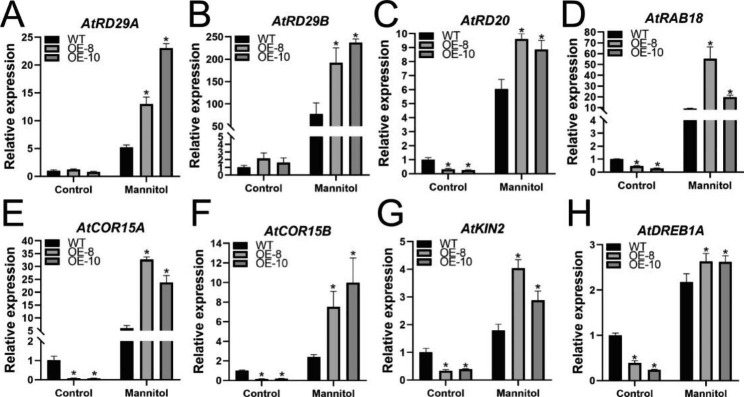
Expression levels of stress-responsive genes in WT and transgenic Arabidopsis plants. **A-H** The transcript levels of *AtRD29A*, *AtRD29B*, *AtRD20*, *AtRAB18*, *AtCOR15A*, *AtCOR15B*, *AtKIN2* and *AtDREB1A* genes under control and simulated drought conditions. Expression values are relative to WT, and data represented means ± SE of three replicates. The asterisk (*) indicated significant differences at *P* < 0.05, as determined by one-way analysis of variance (ANOVA) with Tukey’s post-test

To investigate the mechanisms by which *McWRKY57-like* overexpression enhances drought resistance in Arabidopsis, we further performed qRT-PCR to analyze the expression levels of some stress-responsive genes in transgenic Arabidopsis, including *AtRD29A*, *AtRD29B*, *AtRD20*, *AtRAB18*, *AtCOR15A*, *AtCOR15B*, *AtKIN2*, and *AtDREB1A*. The expression levels of *AtRD29A* and *AtRD29B* were not significantly different between *McWRKY57-like OE* lines and WT plants under normal growth condition but were upregulated in both OE lines and WT plants under mannitol (simulated drought) treatment. However, the increase was more pronounced in *McWRKY57-like OE* lines than in WT plants (Fig. 7A and B). Furthermore, the transcript levels of *AtRD20*, *AtRAB18*, *AtCOR15A*, *AtCOR15B*, *AtKIN2*, and *AtDREB1A* were significantly downregulated in *McWRKY57-like OE* lines compared to WT plants under control conditions. When treated with mannitol, the transcript levels of these genes were evidently upregulated in *McWRKY57-like OE* and WT plants, with markedly higher levels in *McWRKY57-like OE* lines than in WT plants (Fig. 7C–H). The upregulation of these drought-related genes may partly explain the enhanced tolerance of *McWRKY57-like OE* transgenic plants.

## Discussion

Drought stress is a non-negligible factor affecting plant distribution and development. As one of the largest TF families found exclusively in plants, WRKY TFs play pivotal roles in plant growth, development, signal transduction, and responses to abiotic stresses [20, 37]. However, the role of WRKY gene from *M. canadensis* in drought resistance has not been well explored. In this study, we isolated a *McWRKY57-like* gene from *M. canadensis*. Phylogenetic analysis and multiple sequence alignment analysis indicate that McWRKY57-like contains a highly conserved WRKYGQK motif and a C2H2 zinc finger-like motif and is highly homologous to AtWRKY57 (Fig. 1). Thus, it belongs to the WRKY group IIc subfamily. Subcellular localization assay and transcriptional activity assay show that McWRKY57-like is localized in the nucleus and possesses transcriptional activation activity (Fig. 2). *McWRKY57-like* is highly expressed in stems, flowers, rhizome and adventitious roots of mint, suggesting it may play an important role in the development of these organs (Fig. 3A). It was found that GUS staining was undetectable in the roots of *Pro*_*McWRKY57−like*_-*GUS* transgenic Arabidopsis seedlings (Fig. 3E). Root of mint emerges from rhizome, and is called adventitious root; root of Arabidopsis originates from radicle, and is called normal root. We speculate that the difference between mint adventitious root and Arabidopsis root may be one reason of no GUS signal in the roots of transgenic Arabidopsis seedlings. Examination of *McWRKY57-like* expression under simulated drought (mannitol), salt, ABA, and MeJA treatments shows that its expression is most significantly induced by mannitol treatment in both leaves and adventitious roots (Fig. 4). These results suggest that McWRKY57-like, a novel WRKY TF from *M. canadensis*, may play a role in plant response to drought stress.

Growing evidence has demonstrated that WRKY TFs play an increasingly important role in drought stress responses. Previous studies have shown that enhanced *AtWRKY57* expression confers drought tolerance in plants [28, 29]. Overexpression of *TaWRKY2* from wheat enhances drought tolerance of transgenic wheat [38]. The WRKY TF PbrWRKY53 from *Pyrus betulaefolia* plays a positive role in drought tolerance [39]. The sorghum WRKY TF SbWRKY30 functions as a positive regulator in plant response to drought stress [40]. EjWRKY17 from *Eriobotrya japonica* demonstrates a positive role in ABA-regulated drought tolerance [41]. Similarly, we found that *McWRKY57-like* plays a positive role in drought response of transgenic Arabidopsis plants. The size and architecture of the root system are key factors affecting the ability of plants to access water and nutrients, and root length is an important indicator of plant drought tolerance [42–44]. We found that, although the root growth of WT and *McWRKY57-like* OE transgenic seedlings is not significantly different under normal conditions, the root length is significantly longer in *McWRKY57-like OE* transgenic seedlings than in WT seedlings under drought conditions (Fig. 5). The chlorophyll content is a reliable indicator of plant drought tolerance and is positively correlated with drought tolerance [45]. Consistently, chlorophyll content is higher in *McWRKY57-like OE* lines than in WT plants under drought treatment (Fig. 5). The water loss rate is also an important determinant of drought tolerance [24, 46]. We showed that the water loss rate is evidently lower in *McWRKY57-like OE* lines than in WT plants (Fig. 5). These results indicate that overexpression of the *McWRKY57-like* gene may improve water uptake, maintain chlorophyll content stability, and reduce water loss under water-deficit conditions, thus enhancing plant drought tolerance.

When exposed to unfavorable environmental conditions, plants can synthesize some osmoregulatory substances, such as proline, betaine, soluble sugars, and soluble proteins. Among them, proline not only acts as an osmolyte but also functions as a potent antioxidant and programmed cell death inhibitor, making it one of the most important indicators of plant drought tolerance [47, 48]. Soluble sugars and soluble proteins are also important osmoregulatory substances that maintain turgor pressure and macromolecular structure and function, thereby improving plant drought tolerance [43, 44]. In this study, osmotic stresses significantly enhance *McWRKY57-like* expression, and the contents of these three osmolytes are more markedly increased in *McWRKY57-like OE* plants than in WT under drought treatment (Figs. 4 and 6). These results suggest that *McWRKY57-like* overexpression can improve the osmotic regulation ability of plants by accelerating the accumulation of osmoregulatory substances, thereby enhancing the drought tolerance of transgenic plants.

Drought stress can lead to excessive ROS accumulation and disrupt ROS homeostasis, leading to a decline in lipid membrane functions and ultimately to oxidative damage to plant cells [49]. MDA concentration is an important physiological indicator of cell membrane lipid peroxidation and changes in plant under stress [36]. In addition, a series of antioxidant systems, including SOD, CAT, and POD, have been evolved in plants to mitigate ROS-induced damages. A growing number of studies have demonstrated that WRKYs enhance drought tolerance by reducing MDA content or increasing antioxidant enzyme activity in transgenic plants [39, 43, 50]. Our data show that MDA content is significantly decreased, and SOD, CAT, and POD activities are evidently enhanced in *McWRKY57-like OE* plants compared with WT plants after drought treatment (Fig. 6). These results suggest that *McWRKY57-like* can improve ROS scavenging by enhancing antioxidant enzyme activities, thereby improving the drought tolerance of transgenic plants.

WRKY TFs play critical roles in a regulatory network that regulates plant responses to stress by integrating internal and environmental factors via regulating downstream stress-responsive genes [51, 52]. For instance, AtWRKY57 positively regulates the expression of *AtRD29A*, *AtABA3*, and *AtNCED3*, which are responsible for the increased drought tolerance of their overexpression lines [28]. Overexpression of wheat *TaWRKY19* in Arabidopsis enhances its tolerance to salt, drought, and freezing stresses by activating the expression of *AtRD29A*, *AtRD29B*, and *AtDREB2A* [53]. Overexpression of *VaWRKY14* from *Vitis amurensis* in Arabidopsis enhances drought tolerance by upregulating the expression of stress-related genes, such as *AtCOR15A*, *AtCOR15B*, *AtCOR413*, *AtRD29A*, and *AtKIN2* [54]. It has been reported that expression of *AtRD29A* and *AtRD29B* is induced by cold, drought, and salt stress, and these two genes are considered marker genes in response to cold, drought, and salt stress [55]. The expression of *AtRD20* is triggered by water deficiency and plays a positive role in plant drought tolerance [56]. Arabidopsis *RAB18* gene expression is induced to high levels by ABA and drought stress [57]. The cold-responsive genes *AtCOR15A* and *AtCOR15B* are generally upregulated in transgenic Arabidopsis plants, which are more tolerant to drought stress [54, 58]. Arabidopsis *KIN2* gene is strongly responsive to drought and salinity stresses and is considered a drought-responsive marker gene [54, 59, 60]. Overexpression of *AtDREB1A* in transgenic plants activates the expression of stress tolerance genes, thereby increasing tolerance to drought stress [61]. These aforementioned stress-responsive genes are generally considered drought-induced marker genes or ABA-responsive genes [54, 60, 62]. Their expression is upregulated to a higher level in *McWRKY57-like* OE plants than in WT plants under drought conditions (Fig. 7), which may partly explain the enhanced tolerance of *McWRKY57-like* transgenic plants to drought stress. ABA and jasmonate (JA) play crucial roles in the abiotic stress response and development of plants. They can act synergistically to promote stomatal closure and decrease water loss under drought stress [63–65]. In our study, ABA and MeJA treatments significantly enhance *McWRKY57-like* expression (Fig. 4). These results intimate that ABA and JA signaling pathways may play a role in McWRKY57-like-regulated drought tolerance, and this link requires further investigation.

## Conclusions

To summarize, *McWRKY57-like*, a novel WRKY transcription factor gene from *M. canadensis* was reported in this study. The McWRKY57-like protein was a member of group IIc WRKY family, located in nuclear, and had transcription factor activity. The expression of *McWRKY57-like* was highly induced in *M. canadensis* under drought stress. In addition, overexpression of *McWRKY57-like* in Arabidopsis plants significantly enhanced plant drought tolerance. Further investigations revealed that under drought stress, *McWRKY57-like* transgenic plants exhibited higher proline, soluble sugar, and soluble protein contents, higher CAT, SOD, and POD activities, and lower MDA content than WT plants. Moreover, the expression levels of drought-induced maker genes were markedly upregulated in *McWRKY57-like OE* plants than in WT plants under drought stress. The above results indicated that *McWRKY57-like* plays a positive role in plant adaptation to drought conditions by increasing osmosis substance accumulation and antioxidant enzyme activities and regulating the expression of stress-related genes. This study suggests that *McWRKY57-like* may be a valuable genetic resource in molecular breeding programs of plants. Future studies are needed to elucidate the functional mechanism of *McWRKY57-like* against abiotic stresses.

## Methods

### Plant materials and treatments

*M. canadensis* was cultured in a mixture of nutrient soil and vermiculite (2:1, v/v) or in water. Tobacco seeds were sown on soil and 10-day-old seedlings were grown in potting soil. *M. canadensis* and tobacco plants were grown in a growth chamber with a light intensity of 300 µmol m^− 2^ s^− 1^ and a day/night regime of 16/8 h (26 °C). Arabidopsis seeds were sterilized by chlorine and sown onto 1/2 Murashige-Skoog (MS) medium with 1% sucrose and 0.8% agar (w/v), then incubated at 4ºC for 2 days and then transferred to a growth chamber with a light intensity of 200 µmol m^–2^ s^–1^ and a day/night cycle of 14/10 h (22ºC).

For treatments of mannitol, NaCl, ABA and MeJA, 3-week-old water-cultured *M. canadensis* were separately transferred into MS medium containing 300 mM mannitol, 150 mM NaCl, 100 µM ABA, and 200 µM MeJA for 0, 2, 4, 8, 12, and 24 h, and then leaves and adventitious roots were harvested and frozen in liquid nitrogen. For natural drought treatment, 6-week-old mint plants grown in potting soil were stopped to supply water for 10 days, then leaves were collected and frozen in liquid nitrogen. All samples were frozen in liquid nitrogen and stored at − 80 °C for subsequent total RNA isolation.

### Clone and bioinformatics analysis of ***McWRKY57-like*** gene

The coding region of the *McWRKY57-like* gene was amplified from the cDNA of *M. canadensis* using a pair of specific primers. The phylogenetic tree was constructed through the neighbor-joining method on MEGA software with 1000 bootstrap replicates, and the protein sequences of Arabidopsis WRKYs were obtained from PlantTFDB (http://planttfdb.gao-lab.org/index.php). The homologous proteins of McWRKY57-like were searched in Phytozome database (https://phytozome-next.jgi.doe.gov/), and multiple alignments were performed using DNAMAN. The MEME combinatorial tool was used for motif search. The primers used for CDS clone were listed in Table S1.

### Subcellular location of McWRKY57-like

The open reading frame (ORF) of the *McWRKY57-like* gene was amplified and constructed into pGate8-GFP vector to generate *35S::McWRKY57-like-GFP*. The construct was then transformed into *Agrobacterium* strain GV3101. For determination of McWRKY57-like subcellular localization in tobacco leaves, GFP-fused McWRKY57-like protein and nuclear marker were transiently expressed in tobacco leaves using established protocols [66, 67]. To determine the subcellular localization of McWRKY57-like in Arabidopsis seedlings, the construct *35S::McWRKY57-like-GFP* was introduced into WT plants using *Agrobacterium tumefaciens*–mediated transformation via the floral dip method [68], and transgenic plants were confirmed by kanamycin (50 µg/mL) selection and RT-PCR. For DAPI (4’,6-diamidino-2-phenylindole) staining, the samples were incubated with 1 µg/ml DAPI aqueous solution. GFP and red fluorescent protein (RFP) and DAPI imaging in plant tissues were performed using a confocal laser scanning microscope. The excitation/emission wavelengths during acquisition were 488 nm/493–536 nm for GFP, 561 nm/580–650 nm for RFP, and 405 nm/532–632 nm for DAPI. The primers used for *35 S::McWRKY57-like-GFP* construction were listed in Table S1.

### Transcriptional activation assay of McWRKY57-like in yeast cells

The ORF of *McWRKY57-like* was introduced into the pGBKT7 (BD) vector at the site of EcoR1. The BD vector was used as empty control, and pGBKT7-AtSIZ1 (BD-AtSIZ1) [69] was used as positive control. The experimental group pGBKT7-McWRKY57-like (BD- McWRKY57-like), positive control and empty control were separately transformed into Y2H Gold yeast cells using the Yeast Transformation Kit (Coolaber, Beijing, China). The transformed yeast was spotted on SD/-Trp and SD/-Trp/-His/-Ade plates to observe yeast growth at 30℃ for 2–3 days. The primers used for *BD-McWRKY57-like* construction were listed in Table S1.

### RT-PCR and qRT-PCR

Total RNA was extracted from different plant tissues using FastPure Plant Total RNA Isolation Kit (Vazyme) following the manufacturer’s instructions. cDNA obtained via HiScript® III 1st Strand cDNA Synthesis Kit (+ gDNA wiper) (Vazyme) was used for reverse transcription PCR (RT-PCR) and quantitative real-time PCR (qRT-PCR). qRT-PCR was performed using AceQ Universal SYBR qRT-PCR Master Mix (Vazyme) and the CFX96 Real-Time PCR Detection System (Bio-Rad), as specified by the manufacturer. The *McACT* gene was used as the reference gene for normalizing the gene expression in *M. canadensis*, whereas *AtACT2* was used in Arabidopsis. The primers used for RT-PCR and qRT-PCR were listed in supplemental table S1.

### β-Glucuronidase (GUS) staining assay

To generate the *Pro*_*McWRKY57−like*_-*GUS* construct, a 1,224-bp promoter sequence was amplified using the Genome Walking Kit (Takara, Dalian, China) as described by the manufacturers’ instructions, and then cloned into the binary vector PMV2 [70] carrying the *GUS* gene downstream of the inserted promoter. Then, the construct was transformed into Arabidopsis WT plants via the floral dip method [68]. Transgenic plants were screened by kanamycin (50 µg/mL) selection, and the homozygous transgenic lines were used for GUS analysis. GUS histochemical staining was performed by the procedures described previously [71]. GUS-stained tissues were bleached in 75% (v/v) alcohol and photographed using a Leica DVM6a stereoscope. The primers used for amplification of genomic DNA fragments for Genome Walking Kit and construction of *Pro*_*McWRKY57−like*_-*GUS* are listed in Supplementary Table S1.

### Phenotype analysis under drought

For seedling treatments, sterilized seeds of Arabidopsis WT and OE lines were spotted in 1/2 MS medium, and then plated in dark at 4℃ for 2 days followed by vertical placement in a light incubator and grown for 4 days. Then, the uniformed seedlings were transferred to 1/2 MS medium containing 300 mM mannitol and root length was photographed and measured after 7 days of normal incubation. For adult treatments, sterilized seeds were germinated on the 1/2 MS medium for 5 days and planted in the cultivation substrate (soil: vermiculite = 2:1) and incubated in a greenhouse for 3 weeks before natural drought treatment. For natural drought treatment, 3-week-old WT and transgenic plants grown in soil were fully watered first and then stopped watering for 10 days. The plants under normal condition and natural drought treatment were then observed and photographed.

### Measurement of chlorophyll content

3-week-old plants of WT and transgenic plants were treated by natural drought treatment for 10 days. Then, 0.5 g of fresh leaves of each line after normal and drought treatments were collected for chlorophyll extraction. The chlorophyll was extracted using 80% acetone and chlorophyll absorbance was measured by a spectrophotometer under a wavelength of 645 and 663 nm. The 80% acetone solution was used as a blank control and chlorophyll contents were determined by the method described previously [72].

### Measurement of water loss rate

Detached rosette leaves drawing from about 5-week-old plants of WT and transgenic plants grown under normal conditions were used to measure the water loss rates. The leaves were sampled and weighed immediately (fresh weight, FW) and then placed on clean filter paper to dehydrate it naturally under condition of 25℃ and 40–45% relative humidity. Leaf mass was measured at predetermined times, respectively.

### Determination of related physiological indexes

Three-week-old Arabidopsis WT and OE plants after normal and natural drought treatments for 10 days were used for determination of related physiological indexes. The proline, soluble protein, soluble sugar and MDA contents were determined using the Proline assay kit, Plant soluble sugar content test kit, Total protein quantitative assay kit, and the plant Malondialdehyde (MDA) assay kit, respectively. The CAT, SOD and POD activity were measured using the Catalase (CAT) assay kit, Total Superoxide Dismutase (T-SOD) assay kit, and Peroxidase assay kit, respectively. All the kits used above were brought from Nanjing Jiancheng, Nanjing, China, and each physiological index was determined according to the manufacturers’ instructions.

### Analysis of stress-responsive genes

Four-day-old WT and transgenic seedlings grown on 1/2 MS medium were transferred to 1/2 MS medium with and without 300 mM mannitol for normal and simulated drought treatments. After 7-day treatment, seedlings of each line were sampled and used for RNA isolation, and further for confirm the expression of stress-responsive genes, including *AtRD29A*, *AtRD29B*, *AtRD20*, *AtRAB18*, *AtCOR15A*, *AtCOR15B*, *AtKIN2* and *AtDREB1A* by qRT-PCR. The primers used for qRT-PCR are listed in Supplementary Table S1.

## Electronic supplementary material

Below is the link to the electronic supplementary material.

Supplementary Material 1

Supplementary Material 2

## Data Availability

The datasets generated and analysed during the current study are submitted to the NCBI GenBank with submission number of 2,635,888 (McWRKY57-like) and 2,653,970 (McWRKY57-like promoter), and their accession numbers of OP710252 (McWRKY57-like) and OQ082556 (McWRKY57-like promoter) were provided by NCBI. The WRKY transcription factor family of *A. thaliana* are available in PlantTFDB (http://planttfdb.gao-lab.org/family.php?fam=WRKY). The homologous proteins of McWRKY57-like are available in Phytozome database (https://phytozome-next.jgi.doe.gov/) with accession numbers of Solyc05g012500.3.1 (*Solanum lycopersicum*), Gorai.008G178100.1 (*Gossypium raimondii*), Glyma.01G056800.2.p (*Glycine max Wm82*), FvH4_4g30360.t1 (*Fragaria vesca*), At1G69310.1 (*Arabidopsis thaliana*), LOC_Os03g55080.1 (*Oryza sativa*), respectively. The datasets used for BLASTP search of AtWRKY57 are available in the SRA (https://www.ncbi.nlm.nih.gov/sra) repository with a with an accession number of SRP132644. The datasets used and/or analysed during the current study are available from the corresponding author on reasonable request.

## Declarations

### Statement

Plant materials of *Mentha canadensis* L. and *Arabidopsis thaliana* L. were collected from Germplasm Nursery at Nanjing Botanical Garden Mem. Sun Yat-Sen, Nanjing, China. All the required permissions have been obtained from Institute of Botany, Jiangsu Province and Chinese Academy of Sciences. The plant materials don’t include any wild species at risk of extinction. We comply with relevant institutional, national, and international guidelines and legislation for plant study.

## Abbreviations

TF: Transcription factor
bHLH: Basic helix-loop-helix protein
MYB: Myeloblastosis
NAC: NAM, ATAF1/2 and CUC2
DREB: Dehydration responsive element-binding protein
bZIP: Basic region/leucine Zipper
GFP: Green fluorescent protein
qRT-PCR: Quantitative reverse transcription polymerase chain reaction
DAPI: 4′,6‑diamidino‑2‑phenylindole
ORF: Open reading frame
GUS: β-Glucuronidase
MDA: Malondialdehyde
CAT: Catalase
OD: Superoxide dismutase
POD: Peroxidase
