# Supplementary figures and images for: Overexpression of a WRKY transcription factor McWRKY57-like from Mentha canadensis L. enhances drought tolerance in transgenic Arabidopsis
Source: BMC Plant Biol. 2023 Apr 25;23:216. doi: 10.1186/s12870-023-04213-y (PMC10126992; doi:10.1186/s12870-023-04213-y)

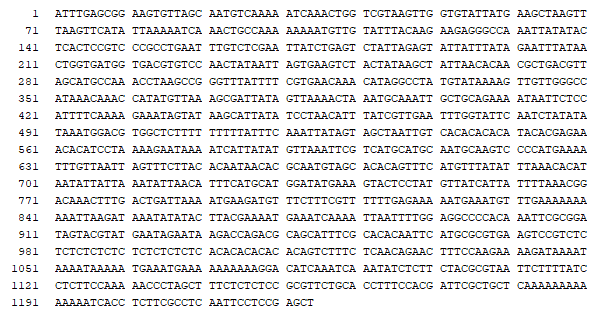


**Figure S1.** Sequence of promoter of *McWRKY57-like* gene.

Supplement: Supplementary file 1 — Supplementary Material 1 [file 12870_2023_4213_MOESM1_ESM.docx]
