# Supplementary material for: Overexpression of a WRKY transcription factor McWRKY57-like from Mentha canadensis L. enhances drought tolerance in transgenic Arabidopsis
Source: BMC Plant Biol. 2023 Apr 25;23:216. doi: 10.1186/s12870-023-04213-y (PMC10126992; doi:10.1186/s12870-023-04213-y)
Supplement: Supplementary file 2 — Supplementary Material 2 [file 12870_2023_4213_MOESM2_ESM.docx]

**Table S1** Details of gene primers used in this article.

| Name |  | Sequence (5’-3’) | Description |
| --- | --- | --- | --- |
| *McWRKY57-like* | Forward | 5’-ATGTCCGGCAAGGAGAAGCC-3’ | CDS clone and RT-PCR |
|  | Reverse | 5’- TCATCCATTATTTCGCATTC-3’ |  |
| *McWRKY57-like OE* | Forward | 5’-GGAGAGGACACGCTCGAGATGTCCGGCAAGGAGAAG-3’ | pGate8-GFP |
|  | Reverse | 5’-CACCATGAATTCCTCGAGTCCATTATTTCGCATTCT-3’ |  |
| *BD-McWRKY57-*  *like* | Forward | 5’-ATGGCCATGGAGGCCGAATTCATGTCCGGCAAGGAGAAG-3’ | pGBKT7 |
|  | Reverse | 5’-TCGACGGATCCCCGGGAATTCTCATCCATTATTTCGCATT-3’ |  |
| *McACTIN* | Forward | 5’-GCTCCAAGGGCTGTGTTCC-3’ | real-time qRT-PCR |
|  | Reverse | 5’-TCTTTCTGTCCCATGCCAAC-3’ |  |
| *McWRKY57-like* | Forward | 5’-CCAAGGAGTTACTATCGATG-3’ | real-time qRT-PCR |
|  | Reverse | 5’-CAATGTTGCCCTTCGTACGT-3’ |  |
| *McWRKY57-likePRO-SP1* |  | 5’-TGAGCAGCGAATCGTGGAAAGG-3’ | promoter amplification |
| *McWRKY57-likePRO-SP1* |  | 5’-GTAATCGTCGCTGCCGAAGAAG-3’ | promoter amplification |
| *McWRKY57-likePRO-SP1* |  | 5’-TTCTGTCCCTTCTTCTTCGTCTTCC-3’ | promoter amplification |
| *Pro_MhWRKY57_-like-GUS* | Forward | 5’-TGGGAGCTCCTCGAGGGTACCATTTGAGCGGAAGTGTTAG-3’ | PMV2 |
|  | Reverse | 5’-AGAGAATTCAAGCTTGGTACCAGCTCGGAGGAATTGAGGC-3’ |  |
| *AtRD29A* | Forward | 5’-TTCTGTAAGGACGACGTTTACA-3’ | real-time qRT-PCR |
|  | Reverse | 5’-CGTACTCGTTACATCCTCTGTT-3’ |  |
| *AtRD29B* | Forward | 5’-GAAACCAAAGATGAGTCGACAC-3’ | real-time qRT-PCR |
|  | Reverse | 5’-TTTTTCGTAAACCGGAGTCAAC-3’ |  |
| *AtRAB18* | Forward | 5’-GGAGGAAGAAGGGAATAACACA-3’ | real-time qRT-PCR |
|  | Reverse | 5’-GGGAAGCTTTTCCTTGATCTTG-3’ |  |
| *AtRD20* | Forward | 5’-CACGATAGCAAAGGAATGAGTG-3’ | real-time qRT-PCR |
|  | Reverse | 5’-TTGAAACCAAGGTCACGAAATC-3’ |  |
| *AtCOR15A* | Forward | 5’-CATTAGCAGATGGTGAGAAAGC-3’ | real-time qRT-PCR |
|  | Reverse | 5’-TCTCAGCTTCTTTACCCAATGT-3’ |  |
| *AtCOR15B* | Forward | 5’-GATGGCGAGAAAACAAAAGACT-3’ | real-time qRT-PCR |
|  | Reverse | 5’-CTTCTGCTTTACCCTCTACGAA-3’ |  |
| *AtKIN2* | Forward | 5’-GCAACAGGCGGGAAAGAGTA-3’ | real-time qRT-PCR |
|  | Reverse | 5’-CCAAAGTTGACTCGGATCGC-3’ |  |
| *AtACTIN2* | Forward | 5’-GACCTTGCTGGACGTGACCTTAC-3’ | real-time qRT-PCR |
|  | Reverse | 5’-GTAGTCAACAGCAACAAAGGAGAGC-3’ |  |
